# Treatment of paradoxical eczematous eruption in psoriasis treated with secukinumab: A case report

**DOI:** 10.1097/MD.0000000000032844

**Published:** 2023-02-10

**Authors:** Yu Xiao, Shanshan Peng, Xiangjun Li, Tianyi Mao, Muping Fang, Youhong Hu, Wenzheng Ye

**Affiliations:** a The Central Hospital of Xiaogan, Xiaogan Hospital Affiliated to Wuhan University of Science and Technology, Hubei, China; b The Central Hospital of Xiaogan, Xiaogan Hospital Affiliated to Wuhan University of Science and Technology, Hubei, China; c The Central Hospital of Xiaogan, Jinzhou Medical University, Hubei, China; d The Central Hospital of Xiaogan, Jinzhou Medical University, Hubei, China; e The Central Hospital of Xiaogan, Xiaogan Hospital Affiliated to Wuhan University of Science and Technology, Hubei, China; f The Central Hospital of Xiaogan, Xiaogan Hospital Affiliated to Wuhan University of Science and Technology, Hubei, China; g The Central Hospital of Xiaogan, Xiaogan Hospital Affiliated to Wuhan University of Science and Technology, Hubei, China.

**Keywords:** eczematous, IL-17A, psoriasis

## Abstract

Eczematous eruption is an increasingly recognized form of drug-related eruption, typically reported in association with interleukin 17 (IL-17)A inhibitors. However, severe paradoxical eczematous eruption due to IL-17A inhibitors has been rarely reported. Herein, we reported a case of a man with severe psoriasis with erythematous scaly plaques on the scalp, trunk, and arms and legs after the administration of secukinumab was initiated.

**Patient concerns:**

We reported a case of a 20-year-old man with severe psoriasis with erythematous scaly plaques on the scalp, trunk, and arms and legs after the administration of secukinumab was initiated. A skin biopsy was performed. It revealed spongiotic dermatitis consistent with eczematous reaction. Direct and indirect immunofluorescence assays were negative.

**Diagnoses:**

He was diagnosed with eczematous eruption.

**Interventions:**

Discontinuation of secukinumab and administration of cyclosporine and prednisone were considered.

**Outcomes:**

Significant improvement was observed, with no adverse events.

**Conclusion:**

Our case shows that eczematous eruption can paradoxically occur in patients on IL-17A inhibitors and this report is expected to increase awareness of the rising number of cutaneous eruptions related to biological agents.

## 1. Introduction

Psoriasis is a chronic, immune-mediated inflammatory cutaneous disorder.^[1]^ Several new biological agents for the treatment of psoriasis, which target the interleukin 17 (IL-17) or IL-12/23 axis, have been discovered.^[1,2]^ For example, secukinumab, a fully human monoclonal antibody, targeting IL-17A has been developed and approved for the treatment of moderate to severe plaque psoriasis.^[3]^ Given the widespread use of secukinumab, its efficacy and safety have been demonstrated in several random clinical trials, wherein several common adverse events, including infection, nasopharyngitis and soreness at the injection site have been reported.^[4]^ However, a few rare side effects, such as atopic dermatitis-like, psoriasis-like, lichenoid eruption have also been reported.^[5,6]^ Herein, we describe a rare case of generalized paradoxical eruption of eczema following the treatment for severe psoriasis with secukinumab.

## 2. Case report

**Figure 1.**
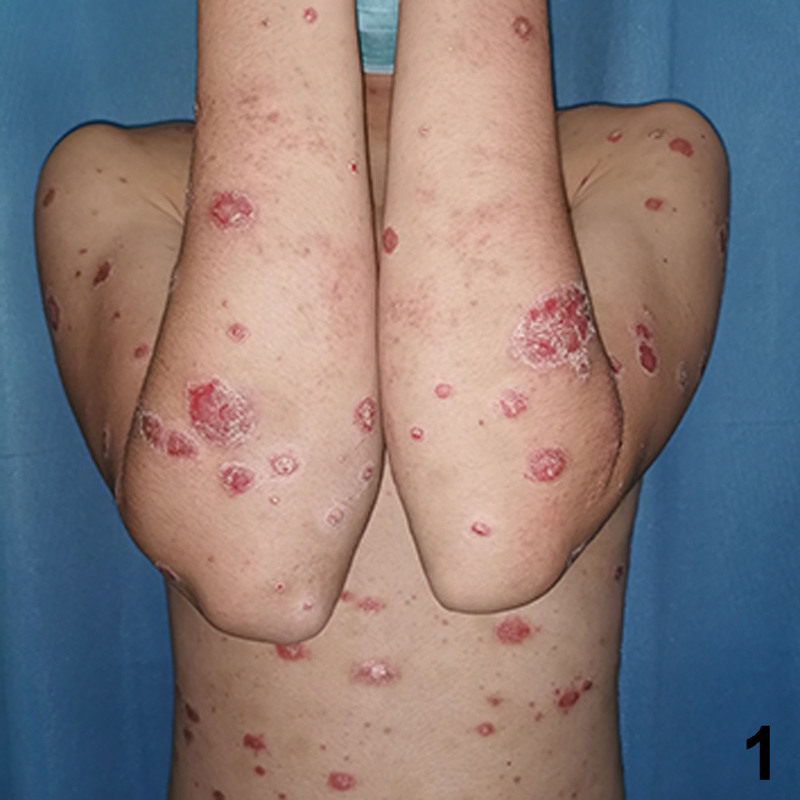
Erythematous scaly plaques on the trunk and arms.

**Figure 2.**
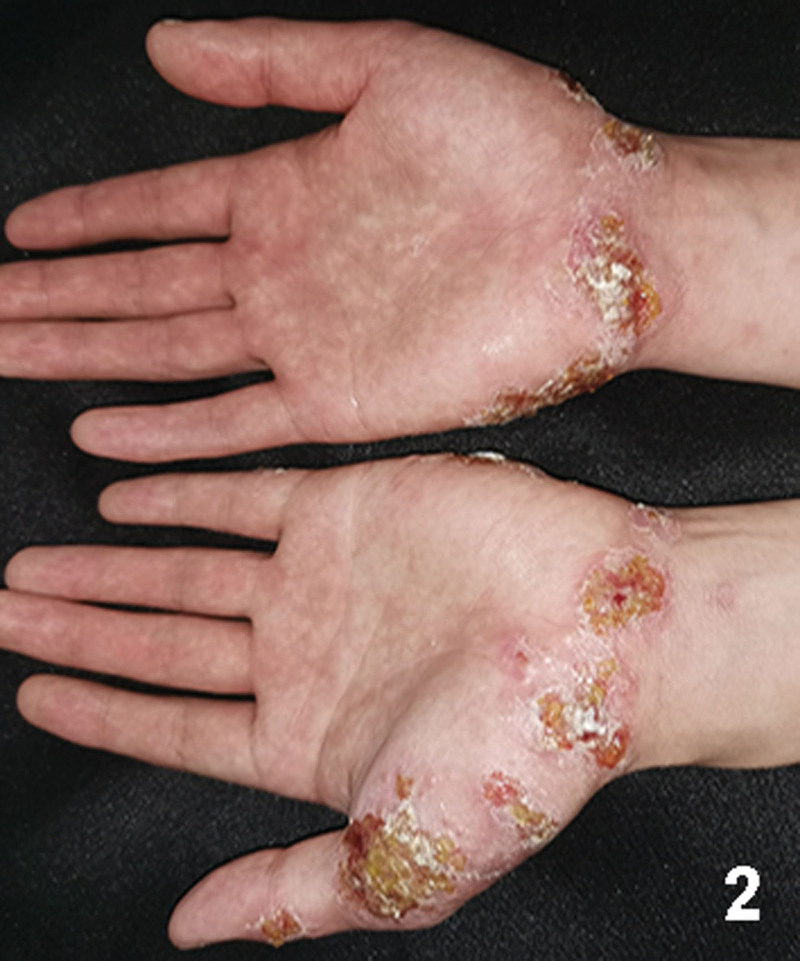
Erythematous eczematous patches and bullous on the dorsal aspects of the hands and palms with superficial excoriations and fissures.

**Figure 3.**
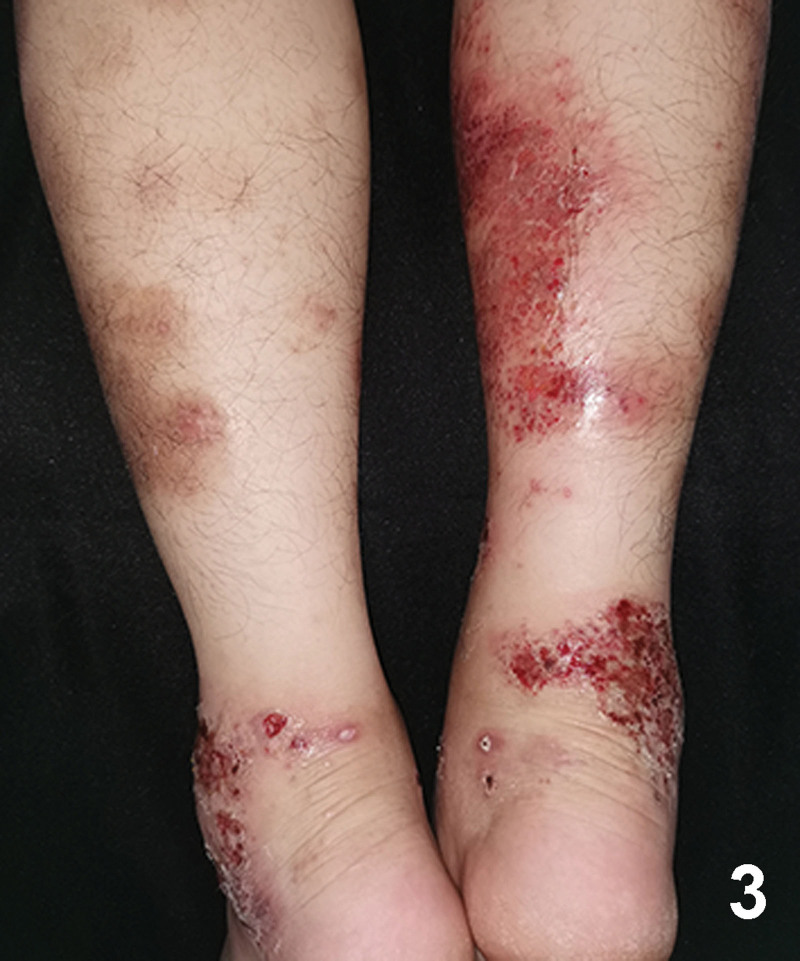
Erythematous eczematous patches and bullous on the flexor aspects of the lower limbs with superficial excoriations and fissures.

**Figure 4.**
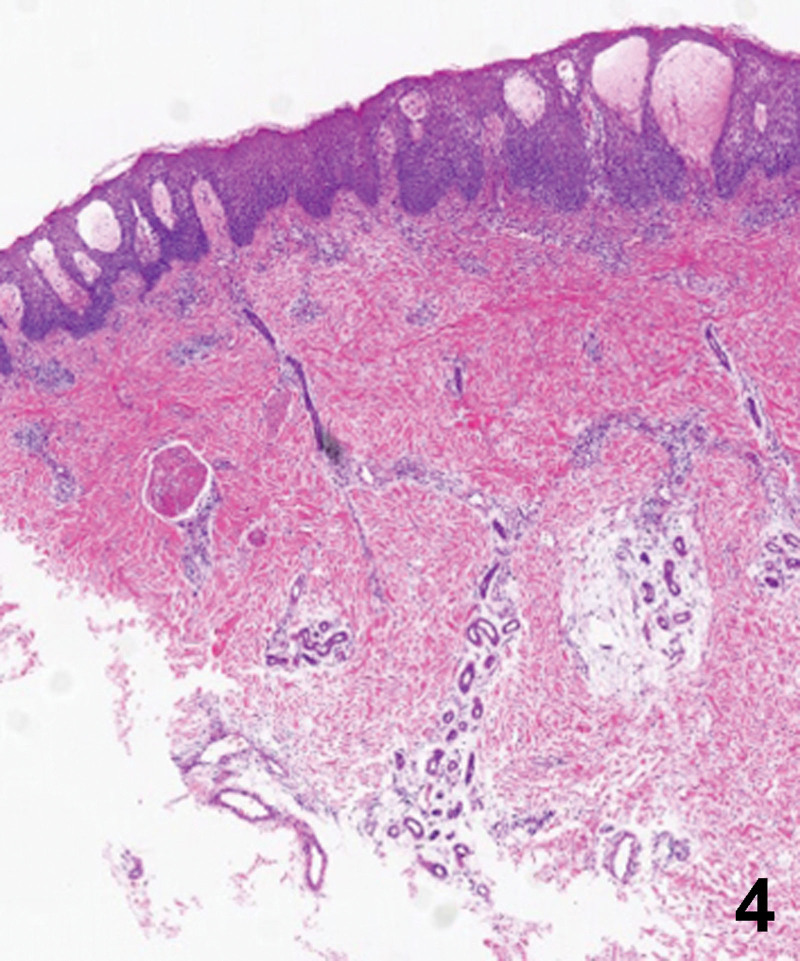
Spongiotic dermatitis with intraepidermal vesicle formation, and superfical and deep perivascular and interstitial inflammation.

**Figure 5.**
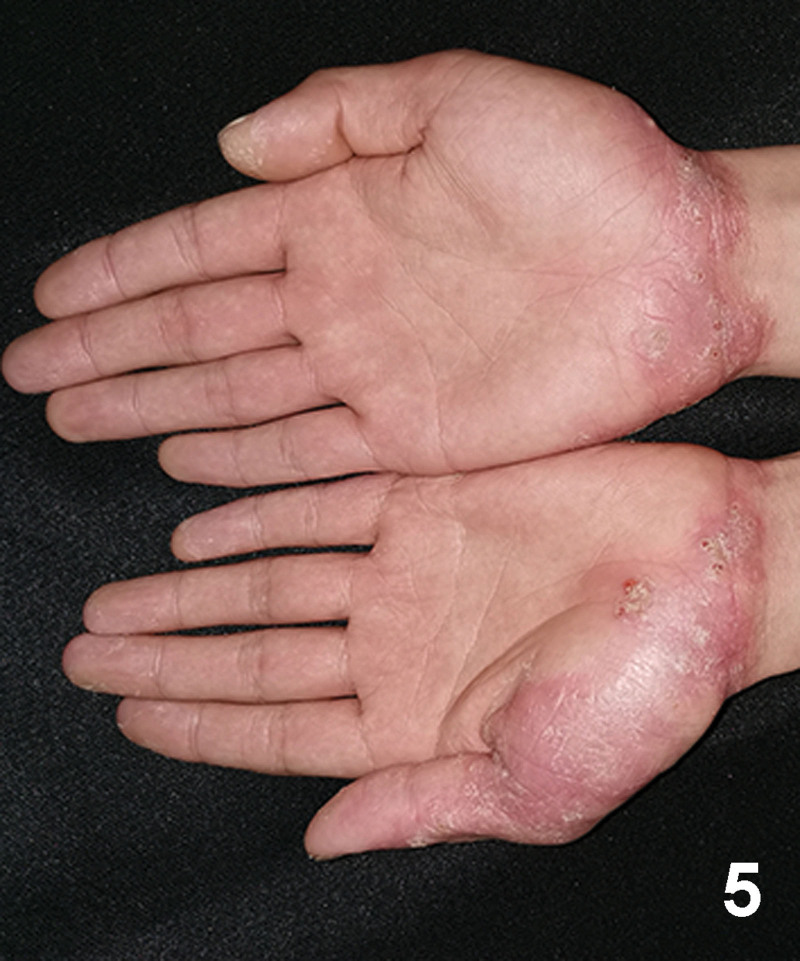
The eruption on the hands showed significant improvement.

**Figure 6.**
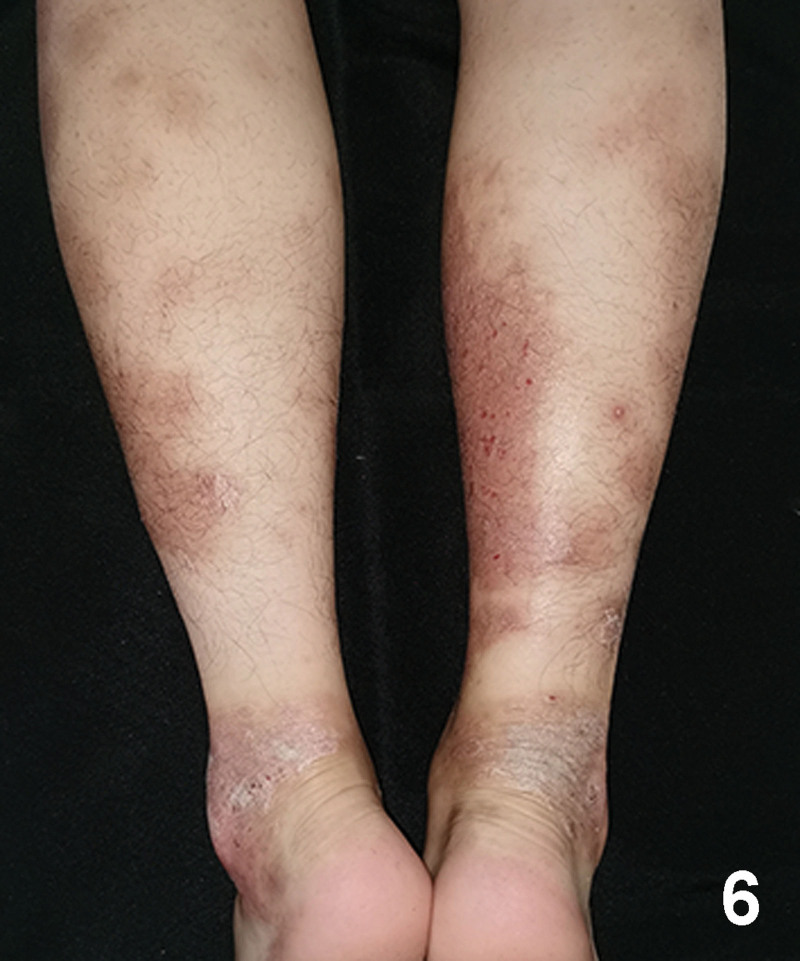
The eruption on the flexor aspects of lower limbs showed significant improvement.

A 20-year-old Chinese male with a 2-year history of psoriasis was referred to a dermatology clinic for psoriasis. Physical examination showed erythematous scaly plaques on the scalp, trunk, and arms and legs covering > 10% of the body surface area (psoriasis area and severity index 20) (Fig. 1). Over the previous year, the lesion showed no improvement with general treatment. Secukinumab 300mg was prescribed to the patient every 1 week and after every week, a maintenance dose of 300mg loading dose for 1 month. Significant improvement in psoriasis was observed at the 4-month follow-up. Two months after secukinumab initiation, the patient presented with new itching lesions on his extremities. Examination revealed erythematous eczematous patches and bullous on the dorsal aspects of the hands and palms with superficial excoriations and fissures, which were in line with eczema (Figs. 2, 3). Treatment with oral loratadine, prednisone and application of topical glucocorticoid creams were prescribed, and injection of 300mg secukinumab was continued. However, new patches and excoriations developed on the trunk on 1week later (eczema area and severity index 25). A skin biopsy was performed. It revealed spongiotic dermatitis consistent with eczematous reaction (Fig. 4). Direct and indirect immunofluorescence were negative. He was diagnosed with eczematous eruption. Discontinuation of secukinumab, and adminstration of cyclosporine (CsA) and prednisone were suggested. An improvement was noted after 2 weeks (Figs. 5, 6). At the time of the manuscript preparation, the patient showed significant improvement with CsA, with no adverse events.

## 3. Discussion

Herein, we describe a case of rare cutaneous side effects of secukinumab, which induces generalized eczematous dermatitis. Psoriasis and eczema are charactrized by distinct T-cell immune responses, whereby T helper 1 (Th1) and Th17 being more prominent in psoriasis, and Th2 in eczema are prominent.^[4]^ The cooccurrence of psoriasis and eczema in the same patient is infrequent due to opposing immune responses. However, several paradoxical cutaneous adverse effects have been demonstrated during the biological treatment for psoriasis and eczema.^[4,7,8]^ For instance, dupilumab, targeting the IL-4 receptor alpha subunit, inhibits the IL-4 and IL-13 signaling cascades, thereby inducing the Th1 or Th17 immune-associated cutaneous diseases during in treatment of AD.^[9]^ Similarly, Th2 immune-associated cutaneous disorders have been reported in biogical treatement of psoriasis. IL17 plays a vital role in the mucoepithelial defenses against *Candida* infections.^[4]^ Moreover, studies on mouse model suggest that IL-17A has a significant role in maitaining the skin microbiome homeostasis and in the expression of filaggrin.^[4,10]^ Consequently, several researchers speculated that preventing Th1 and Th17 cytokines could induce an imbalance in Th2 prominent immune responses. In this case, IL-17A therapy likely prevented the Th1/Th17 phenotype of psoriasis, skewing the balance to a Th2 immune-associated phenotype feature of eczema, evidenced by the development of generalized eczematous dermatitis.^[4]^ Additionally, and IL-22 and IL17C have crucial roles in the development of eczematous dermatitis.^[10]^

Eczematous dermatitis is induced by the treatment with tumor necrosis factor-alpha inbibitors in individuals with psoriasis.^[11]^ Interestingly, an atopic history is a predicitive factor for TNF*α*inhibitor induced eczematous dermatitis.^[11]^ Similarly, all psoriasis patients, developing eczematous dermatitis during the treatment with IL17 inhibitors showed an atopic history, and suggesting that the atopic history serves as a predictive factor for subsequent biologic treatment.^[4]^ However, atopic history was not discernable in our case. Consequently, atopic history may not be a single predictive factor.

There is an urgent need for the treatment of eczematous dermatitis due to psoriasis biologics. In the majority of the previous cases, clinicians withdrew psoriasis biologics after minimal response to topical and systemic steroids.^[4,8]^ In our case, the patient showed almost no improvement with various eczematous dermatitis treatments and gradually new lesions were induced. Generalized eczematous dermatitis presented a good response to CsA and systemic steroids after the discontinuation of secukinumab. At the time of manuscript preparation, our patient showed complete remission with no replase.

Our case suggests that IL17A inhibitor treatment may be a culprit agent for eczematous dermatitis in psoriasis patients. Dermatologists should consider these rare cutaneous adverse effects while prescribing treatment with biologics.

## Author contributions

**Conceptualization:** Shanshan Peng.

**Data curation:** Xiangjun Li, Wenzheng Ye.

**Formal analysis:** Xiangjun Li.

**Funding acquisition:** Yu Xiao.

**Investigation:** Yu Xiao, Tianyi Mao.

**Project administration:** Yu Xiao, Shanshan Peng.

**Resources:** Yu Xiao, Tianyi Mao, Wenzheng Ye.

**Software:** Tianyi Mao, Muping Fang.

**Supervision:** Yu Xiao, Muping Fang, Youhong Hu, Wenzheng Ye.

**Validation:** Shanshan Peng, Muping Fang.

**Visualization:** Yu Xiao, Shanshan Peng, Muping Fang.

**Writing – original draft:** Yu Xiao, Shanshan Peng, Youhong Hu.

**Writing – review & editing:** Yu Xiao, Youhong Hu, Wenzheng Ye.

## References

[1] Griffiths CEM Armstrong AW Gudjonsson JE. Psoriasis. Lancet (London, England). 2021;397:1301–15. 33812489 10.1016/S0140-6736(20)32549-6

[2] Al-Janabi A Foulkes AC Mason K. Phenotypic switch to eczema in patients receiving biologics for plaque psoriasis: a systematic review. J Eur Acad Dermatol Venereol. 2020;34:1440–8. 31997406 10.1111/jdv.16246

[3] Bernardini N Skroza N Tolino E. IL-17 and its role in inflammatory, autoimmune, and oncological skin diseases: state of art. Int J Dermatol. 2020;59:406–11. 31663126 10.1111/ijd.14695 PMC7216999

[4] Caldarola G Pirro F Di Stefani A. Clinical and histopathological characterization of eczematous eruptions occurring in course of anti IL-17 treatment: a case series and review of the literature. Expert Opin Biol Ther. 2020;20:665–72. 32045273 10.1080/14712598.2020.1727439

[5] Mendes Roncada EV Brambilla VR Freitas Filitto B. Atopic dermatitis as a paradoxical effect of secukinumab for the treatment of psoriasis. Case Rep Dermatol. 2021;13:336–9. 34326727 10.1159/000513467 PMC8299393

[6] Lai FYX Higgins E Smith CH. Morphologic switch from psoriasiform to eczematous dermatitis after Anti-IL-17 therapy: a case series. JAMA Dermatol. 2019;155:1082–4. 31290949 10.1001/jamadermatol.2019.1268

[7] Blum AE Burgin S. Eczematous drug eruptions. Am J Clin Dermatol. 2021;22:349–66. 33587283 10.1007/s40257-021-00586-8

[8] Koschitzky M Tan K Noliza Encarnacion MR. Eczematous reactions to psoriasis biologics treated with dupilumab: a case series. JAAD Case Rep. 2021;11:29–32. 33898680 10.1016/j.jdcr.2021.03.006 PMC8058610

[9] Wollenberg A Barbarot S Bieber T. Consensus-based European guidelines for treatment of atopic eczema (atopic dermatitis) in adults and children: part II. J Eur Acad Dermatol Venereol. 2018;32:850–78. 29878606 10.1111/jdv.14888

[10] Megna M Caiazzo G Parisi M. Eczematous drug eruption in patients with psoriasis under anti-interleukin-17A: does interleukin-22 play a key role? Clin Exp Dermatol. 2022;47:918–25. 34862807 10.1111/ced.15052

[11] Deubelbeiss C Kolios AGA Anzengruber F. TNFalpha and IL-17A are differentially expressed in psoriasis-like versus eczema-like drug reactions to TNFalpha antagonists. J Cutan Pathol. 2018;45:23–8. 29023827 10.1111/cup.13055

